# Transient Dynamics of Archaea and Bacteria in Sediments and Brine Across a Salinity Gradient in a Solar Saltern of Goa, India

**DOI:** 10.3389/fmicb.2020.01891

**Published:** 2020-08-13

**Authors:** Kabilan Mani, Najwa Taib, Mylène Hugoni, Gisele Bronner, Judith M. Bragança, Didier Debroas

**Affiliations:** ^1^Department of Biological Sciences, Birla Institute of Technology and Science Pilani, K K Birla Goa Campus, Zuarinagar, India; ^2^Center for Molecular Medicine & Therapeutics, PSG Institute of Medical Sciences and Research, Coimbatore, India; ^3^UMR CNRS 6023, Laboratoire Microorganismes: Génome et Environnement (LMGE), Université Clermont Auvergne, Clermont-Ferrand, France; ^4^Univ Lyon, Université Claude Bernard Lyon 1, CNRS, INRAE, VetAgro Sup, UMR Ecologie Microbienne, Villeurbanne, France

**Keywords:** hypersaline environments, solar saltern, microbial diversity, metabarcoding, archaea, bacteria

## Abstract

The microbial fluctuations along an increasing salinity gradient during two different salt production phases – initial salt harvesting (ISH) phase and peak salt harvesting (PSH) phase of Siridao solar salterns in Goa, India were examined through high-throughput sequencing of 16S rRNA genes on Illumina MiSeq platform. Elemental analysis of the brine samples showed high concentration of sodium (Na^+^) and chloride (Cl^–^) ions thereby indicating its thalassohaline nature. Comparison of relative abundance of sequences revealed that Archaea transited from sediment to brine while Bacteria transited from brine to sediment with increasing salinity. Frequency of Archaea was found to be significantly enriched even in low and moderate salinity sediments with their relative sequence abundance reaching as high as 85%. *Euryarchaeota* was found to be the dominant archaeal phylum containing 19 and 17 genera in sediments and brine, respectively. Phylotypes belonging to *Halorubrum*, *Haloarcula*, *Halorhabdus*, and *Haloplanus* were common in both sediments and brine. Occurence of *Halobacterium* and *Natronomonas* were exclusive to sediments while *Halonotius* was exclusive to brine. Among sediments, relative sequence frequency of *Halorubrum*, and *Halorhabdus* decreased while *Haloarcula*, *Haloplanus*, and *Natronomonas* increased with increasing salinity. Similarly, the relative abundance of *Haloarcula* and *Halorubrum* increased with increasing salinity in brine. Sediments and brine samples harbored about 20 and 17 bacterial phyla, respectively. *Bacteroidetes*, *Proteobacteria*, and *Chloroflexi* were the common bacterial phyla in both sediments and brine while *Firmicutes* were dominant albeit in sediments alone. Further, *Gammaproteobacteria*, *Alphaproteobacteria*, and *Deltaproteobacteria* were observed to be the abundant class within the *Proteobacteria*. Among the bacterial genera, phylotypes belonging to *Rubricoccus* and *Halomonas* were widely detected in both brine and sediment while *Thioalkalispira*, *Desulfovermiculus*, and *Marinobacter* were selectively present in sediments. This study suggests that Bacteria are more susceptible to salinity fluctuations than Archaea, with many bacterial genera being compartment and phase-specific. Our study further indicated that Archaea rather than Bacteria could withstand the wide salinity fluctuation and attain a stable community structure within a short time-frame.

## Introduction

Hypersaline environments like salt lakes and solar salterns are characterized by the presence of high content of salt (>3.5% salinity) and therefore the diversity of organisms in these habitats is unique (Oren, 2002; Ventosa, 2006; Paul and Mormile, 2017). Halophilic organisms counter the high extracellular salinity by two different strategies. In the first strategy, Archaea and a few Bacteria (members exclusively belonging to order *Halanaerobiales* and genus *Salinibacter*) have adapted the “salt-in” strategy of accumulating K^+^ and Cl^–^ ions while maintaining low Na^+^ concentration in response to the external osmotic pressure. In the second strategy, most Bacteria and Eukaryotes combat high salinity by accumulating or synthesizing organic solutes like glycine betaine, ectoine, and other derivatives of amino acids and sugars (Oren, 2002, 2013; Roberts, 2005). Apart from high salinity, these ecosystems further experience fluctuations in pH and temperature, facilitating, polyextremophilic organisms like halophilic alkaliphiles and halophilic alkalithermophiles to inhabit (Grant et al., 1999; Bowers and Wiegel, 2011; Mesbah and Wiegel, 2012).

Coastal solar salterns, employed primarily for the production of edible sodium chloride, contain a series of pans for concentrating seawater, thereby facilitating the sequential precipitation of calcite (CaCO_3_), gypsum (CaSO_4_) followed by halite (NaCl), leaving behind salts of magnesium and potassium (Javor, 2002). These ecosystems make an excellent model for studying the diversity patterns due to the maintenance of constant salinity over a period with minimal external disturbances. Further, the predictable chemistry and limited biodiversity of these systems make the coastal solar salterns an ideal platform for scaling the studies on the evaporative sequence of minerals, biological composition and activity over a broad range of salinities to much complex hypersaline ecosystems (Benlloch et al., 2002; Oren, 2009). In particular, studies on hypersaline environments have gathered interest in the past four decades (Rodriguez-Valera et al., 1981; Ventosa et al., 1982, 1998) due to their importance in understanding the metabolic, physiological and genetic adaptation of organisms in high salinity coupled with their potential biotechnological applications (Oren, 1999, 2010; Siglioccolo et al., 2011; Yin et al., 2015; Gunde-Cimerman et al., 2018).

Culture-independent studies carried out so far in assessing the prokaryotic diversity of coastal and inland solar salterns have identified that the relative sequence abundance of primary bacterial phyla distributed in low salinity samples (4–7%) were *Proteobacteria*, *Cyanobacteria*, *Firmicutes*, *Bacteroidetes*, *Actinobacteria*, and *Tenericutes*, a community composition similar to marine environments. In the intermediate salinity samples (13–18%), bacterial phylotypes affiliated to phyla *Proteobacteria*, *Bacteroidetes*, *Actinobacteria*, and *Verrumicrobia* were usually encountered while the archaeal members belonged to genera *Halorubrum*, *Haloarcula*, and *Haloquadratum* (phylum *Euryarchaeota*). High salinity samples (>25%) were dominated by halophilic archaea belonging to genera such as *Haloquadratum* and *Halorubrum* along with a minor contribution from bacterial phylotypes belonging to *Bacteroidetes* (genus *Salinibacter*) (Mouné et al., 2003; Pašić et al., 2005; Sørensen et al., 2005; Park et al., 2006; Wang et al., 2007; Baati et al., 2008, 2010; Tsiamis et al., 2008; Manikandan et al., 2009; Oren et al., 2009; Oh et al., 2010; López-López et al., 2010; Zafrilla et al., 2010; Tkavc et al., 2011; Trigui et al., 2011; Boujelben et al., 2012; Dillon et al., 2013; Fernandez et al., 2014a, b; Mani et al., 2015; Çinar and Mutlu, 2016; Di Meglio et al., 2016; Zhang et al., 2016; Kalwasińska et al., 2018; Leoni et al., 2020). In general, archaeal composition from the total prokaryotic community was observed to be between 27–46% at intermediate salinities (13–19%) and 80–90% at high salinities (>25%) (Ghai et al., 2011; Fernandez et al., 2014a, b). Though the general prokaryotic community distribution pattern has been established, a majority of these studies have employed techniques that suffer from limited phylogenetic resolution, like clone library analysis of 16S rRNA sequences or denaturing gradient gel electrophoresis (DGGE) and hence the data available on halophilic 16S rRNA gene diversity is still limited. Further, these studies have focused on the prokaryotic diversity of either brine or sediments and therefore a clear picture on the transition of the prokaryotic community between brine and sediments, through an increasing salinity from (∼5 to ∼37%) is still lacking. In the present work we have employed amplicon sequencing of 16S rRNA genes to study the dynamics of prokaryotic community composition of brine and sediment samples obtained from an artisanal solar saltern located in Goa, India at two different time periods of salt production.

Goa is a coastal state in India, having several solar salterns. These coastal salterns are located predominantly along the estuaries, due to easy access to seawater during tidal influxes. Goa receives an annual rainfall of 280–480 cm, maximally from the month of June to November thereby ceasing salt production. The salt pans are constructed in the months of December and January (preparatory phase) and salt production starts from February and continues till May. In the month of February, the salterns would begin the operation through the construction of compartments that are designated into reservoir pan (RP), evaporator pan (EP) and crystallizer pan (CP). Salt crystals produced in the month of February are usually not harvested and rather allowed to settle in the crystallizer pan forming a hard bed of salt crystals thereby facilitating the collection of clean salt without any impurities. This phase lasts for a period of 20 – 25 days and is termed as initial salt harvesting (ISH) phase. Due to the prevailing climatic conditions (low temperature and less wind) and low salinity of inlet water (2%), the time required for salt crystallization varies from 4 to 5 days. However, by March, with the onset of summer, compounded by strong winds and ample sunshine, the time required for salt crystallization in CP decreases to a day or two. This phase of salt production lasts until May and constitutes the peak salt harvesting (PSH) phase. This entire process is repeated on an annual basis and therefore the microbes in this ecosystem are subjected to varying salinity from 2% to 30%. This salinity fluctuation makes the salterns of Goa an ideal candidate for studying the microbial tolerance toward widely fluctuating salinities. The described salt production process is in contrast with the majority of solar salterns located elsewhere around the world, where the brine retention time normally varies from months to years compared to the salterns investigated in this study (Mani et al., 2012b).

In this study, we aimed at investigating the microbial community response toward sudden onset and sustained salinity over a time scale by profiling the prokaryotic composition of a coastal solar saltern between ISH and PSH. Given the nature of solar salterns of Goa, it was hypothesized that Archaea might be more susceptible to changes in the salinity fluctuations prevalent in solar salterns in comparison to Bacteria. Further, Bacteria were expected to dominate the salterns during the ISH and Archaea to dominate during the PSH phase owing to the sustained high salinity. Previous studies conducted in vertical depths of marine environments (Feng et al., 2009; Hamdan et al., 2013; Walsh et al., 2016) have shown that the prokaryotic community composition in water and sediment samples are distinct. Additionally, a study carried out by Walsh et al. (2016) suggested that water could act as a carrier in transporting the microbes over geographical distances due to the presence of rare OTUs in water, otherwise abundant in sediments. Therefore, we also sought to investigate the similarity in the community composition of brine and sediment samples since the compartments of salterns are interconnected and there is a constant influx of brine along a salinity gradient.

## Materials and Methods

### Sample Collection

Brine and surface sediments were collected from solar salterns (15°44′N, 73°86′E) located at Siridao in Goa, India (Supplementary Figure S1). Sampling was carried out in defined compartments namely RP, EP, and CP. Reservoir pans (RP) are primarily used for storing seawater during the tidal influxes. Once the salinity reaches around 5%, the brine is released into the evaporator pans (EP), where the brine is further concentrated until it reaches 23–25% salinity. When the salinity in the EP crosses 5% and 13%, calcium carbonate (CaCO_3_) and calcium sulfate (CaSO_4_) precipitates, respectively. This concentrated brine is finally fed to the crystallizer pans (CP) where the sodium chloride (NaCl) crystallizes around 28% salinity leaving behind magnesium (Mg^2+^) and potassium (K^+^) (Mani et al., 2012b). About 500 ml of Brine samples were collected in a sterile borosilicate glass bottles from the middle of the pans at a depth of 5–10 cm. Similarly, about 50 g of sediments were scooped using a sterile spatula at a depth of 0–5 cm from the surface and stored in polyethylene bags. Both brine and sediment samples were transported in ice and stored at −20°C. Replicate sampling (*n* = 5) was carried out at each sampling point and a composite sample representing each pan was prepared by mixing these replicates into a single sample. A similar approach was adopted for a simultaneous sampling of brine that was subjected to elemental analysis with exceptions of restricting the number of replicates to 3 and storing the samples at 4°C. Initial sampling was carried out in the month of February 2014, during the ISH phase and the second sampling in the month of May 2014 during the PSH phase.

### Physico-Chemical Parameter Estimation and Elemental Analysis

The salinity of the brine samples was measured using a Baumé hydrometer while pH and temperature were documented onsite using a portable pH/temperature meter (Equiptronics, India). Prior to the salinity measurements, Baumé hydrometer was standardized against distilled water at 0% salinity, with reading corresponding to 0 Bé. Salinity and pH of the sediments were measured from the extracts obtained from 1:5 sediment: water mixture. The brine samples were subjected to elemental analysis after filtering through a 0.22 μm filter and diluting them appropriately. The concentration of monovalent cations, sodium (Na^+^) and potassium (K^+^) prevalent in the brine samples was measured through flame emission photometry (Systronics, India). Calcium (Ca^2+^) and magnesium (Mg^2+^) concentration were determined simultaneously by complexometric titration against ethylenediaminetetraacetic acid (EDTA) in the presence of Eriochrome Black T as an indicator (De Sousa, 1954). Chloride (Cl^–^) ion content was determined by argentometric titration against silver nitrate solution in the presence of potassium chromate as an indicator (Skoog et al., 1996).

### Environmental DNA Extraction and Amplicon Sequencing of 16S rRNA Genes

The nucleic acid extraction protocol involved a combination of mechanical, enzymatic and chemical lysis of microbial cells (Zhou et al., 1996). About 5 g of sediment was washed thrice with 100 mM sodium phosphate buffer (pH 7.0)followed by bead beating in the presence of extraction buffer (100 mM tris hydrochloride (pH 8.0), 100 mM ethylenediaminetetraacetic acid (pH 8.0), 100 mM sodium phosphate (pH 8.0), 1.5 M sodium chloride, 2.5% cetyltrimethylammonium bromide and 1% polyvinylpyrrolidone). In the case of brine samples, about 200 mL was vacuum-filtered through a 0.22 μm polycarbonate filter and the obtained membrane was treated similarly to sediment samples. Following the addition of extraction buffer, samples were incubated at 65°C for 2 h after the addition of proteinase K (10 mg/ml) and 20% sodium dodecyl sulfate (SDS). After incubation, DNA was extracted with chloroform:isoamylalcohol (24:1 vol/vol) and precipitated with isopropanol. Obtained crude DNA extracts from brine and sediments were purified using PowerClean DNA Clean-Up Kit (MoBio Laboratories, United States).

Amplification of the V4-V5 region of the 16S rRNA genes was performed using universal prokaryotic primers 515F (5′-GTG YCA GCM GCC GCG GTA-3′) and 909R (5′-CCC CGY CAA TTC MTT TRA GT-3′) (Wang and Qian, 2009; Tamaki et al., 2011). Each reaction mixture contained 10 X PCR buffer, 10 mM of each dNTPs, 2 mM MgCl_2_, 1U Taq Polymerase (ThermoFischer Scientific, United States), 10 mM primers (8-base barcoded), 8 μg BSA and 10 ng template DNA. PCR reactions were performed in duplicates with the following conditions: initial denaturation at 94°C for 10 min followed by 35 cycles of denaturation at 94°C for 60 s, annealing at 58°C for 60 s, elongation at 72°C for 90 s and a final extension at 72°C for 10 min. The PCR products were purified with MinElute kit (Qiagen, United States) and quantified using Qant-iT PicoGreen dsDNA Assay Kit (ThermoFischer Scientific, United States). The amplicons were normalized and multiplexed followed by library preparation and sequencing with 300-bp paired-end sequencing protocol on the Illumina MiSeq platform (Illumina, United States) at GenoScreen, France.

### Bioinformatics and Statistical Analysis of 16S rRNA Amplicon Sequencing Data

Reads generated from the Illumina sequencing platform were processed sequentially using the PANAM pipeline^1^ (Hugoni et al., 2013; Taib et al., 2013). Quality processing steps involved removing reads containing bases with ambiguous nucleotides (N) and PHRED quality score <20. Further, reads with the length shorter than 200 bp and a mismatch in the forward primer sequence were also removed. This was followed by merging the paired-end reads using PANDAseq with a threshold value of 95% (Masella et al., 2012). Following the cleaning procedure, reads were checked for chimera using UCHIME and the clustering of the quality checked reads was carried out using UCLUSTwith a threshold value of 97% (Edgar, 2010). The final step involved taxonomic assignment by the phylogenetic affiliation procedure of PANAM. Briefly, the seed OTUs were compared against reference sequences obtained from the SSURefSILVA database using USEARCH followed by appending each sequence to a phyletic group along with five best hits and sorting the query sequences according to their assignment. Further, HMMalign was used to align the homologous reads with reference sequences from the corresponding profile and a phylogenetic tree was constructed for each profile with FASTTREE2 using Jukes-Cantor + Cat model and with a bootstrap threshold of 100 replicates. Following the phylogenetic tree construction, files containing the details of the taxonomy of bacterial and archaeal sequences (inferred by the lowest common ancestor) and their nearest neighbor were obtained. Alpha and Beta diversity analysis was performed after removing the singletons from the analyzed reads and randomly normalizing to 4100 sequences. The obtained rarefied reads were used to plot rarefaction curves depicting the sampling effort. Alpha diversity measures like species diversity (Shannon index), richness (Chao1), abundance-based coverage estimator (ACE), dominance, and evenness indices were calculated. Diversity indices like Shannon, Chao1, ACE and plotting of rarefaction curves were automated with PANAM. The remaining alpha diversity measures viz. Dominance and Buzas-Gibson’s evenness indices were estimated using PAST (PAlaeontologicalSTatistics) (Hammer et al., 2005). The overall similarity among the microbial community composition between ISH and PSH was analyzed based on Bray-Curtis dissimilarities and visualized using Non-Metric Dimensional Scaling (NMDS) (Minchin, 1987) in vegan package (Oksanen et al., 2013) implemented in R (R Development Core Team, 2014). Statistically significant variation in microbiome taxonomic composition was assed using analysis of similarity (ANOSIM) function in vegan. Unique and common operational taxonomic units (OTUs) shared between different sampling sites were identified through plotting Venn diagrams using jvenn (Bardou et al., 2014).

## Results

### Physico-Chemical Characteristics of the Saltern

The salinity of brine samples increased from 2.1% to 25.2% and 4% to 28% during ISH and PSH respectively (Table 1). Similarly, the salinity of sediment samples increased from 1.3% to 22.3% and 2.3% to 24.8% during ISH and PSH, respectively. The pH of sampling sites varied from 6.5 to 7.8 indicating that the samples are near neutral. The mean temperature of sampling sites during the ISH and PSH phase was 33°C and 38°C, respectively. Though some differences between the temperatures could be observed, the temperature profile of the salterns remained relatively stable throughout the operational stages, a typical feature of the tropical climatic zone (Couto-Rodríguez and Montalvo-Rodríguez, 2019). Elemental analysis of the brine samples indicated that the salterns are dominated with sodium and chloride ions indicating the thalassohaline nature. Most cations (sodium, magnesium and potassium) except for calcium and anion (chloride) increased with increasing salinity (Table 1). A decrease in the concentration of calcium in CP when compared with EP during both phases of salt production could be attributed to the precipitation of gypsum (calcium sulfate) in the intermediate salinity which was apparent during the sampling.

**TABLE 1:** Physico-chemical parameters of brine and sediments^a,b^ obtained from various compartments of solar saltern.

Sampling sites^c^	pH	Temp. (°C)	Salinity (%)	Na^+^ (g l^–1^)	K^+^ (g l^–1^)	Mg^2+^ (g l^–1^)	Ca^2+^ (g l^–1^)	Cl^–^ (g l^–1^)
**ISH**								
RP	7.1	33.2	2.1	9.9	0.4	0.2	0.5	18.4
EP	6.5	32.9	13.6	35.19	1.1	0.4	1.3	65.51
CP	7.4	33.4	25.2	68.2	4.2	8.6	0.3	119.28
**PSH**								
RP	7.2	38.2	4	10.8	0.4	0.2	0.7	19
EP	7.6	38.4	17.4	45.02	2.2	0.3	1.1	85.44
CP	7.8	38.1	28	72.45	4.5	8.6	0.3	141.8

*^*a*^Salinity of sediment samples in (%): ISH – RP – 1.3%; EP – 9.1%; CP – 22.3%; PSH – RP – 2.3%; EP – 13.5%; CP – 24.8%. ^*b*^Elemental analysis was not carried out for the sediment samples. ^*c*^ISH and PSH phase sampling was carried out in the months of February and May 2014, respectively.*

### Diversity Patterns of the Solar Saltern

**FIGURE 1:**
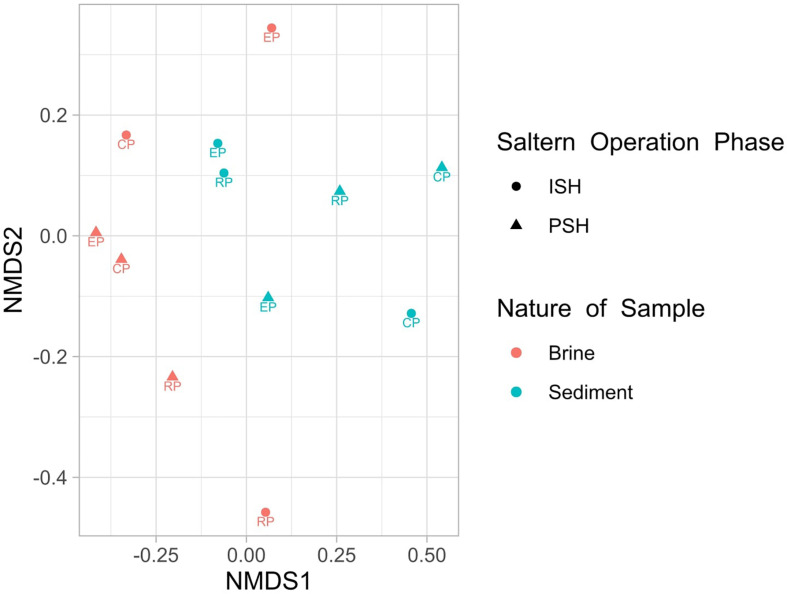
Non-metric Multidimensional Scaling (NMDS) plot showing similarity in the microbial composition (Bray–Curtis distance) of solar salterns during ISH and PSH phase of salt production. RP stands for reservoir pan, EP for evaporator pan and CP for crystallizer pan.

Quality filtering and processing the reads pertaining to the V4-V5 region of the 16S rRNA gene generated 403010 and 2930653 sequences corresponding to Bacteria and Archaea, respectively. Further, taxonomic affiliation with 97% similarity threshold yielded 2638 and 6681 OTUs related to bacterial and archaeal phylotypes respectively (Table 2). On comparing the archaeal and bacterial OTUs within the same sampling sites, it was observed that Archaea had at least two-fold higher OTUs than Bacteria. Similarly, when the OTUs that are commonly distributed across multiple sampling sites were analyzed, it was again observed that bacterial OTUs were outnumbered by archaeal OTUs. This indicated that the similar archaeal phylotypes were dwelling along an increasing salinity gradient in multiple sampling sites (Supplementary Figure S2). On a further note, only 17 OTUs were present consistently across all sampling sites and out of which, 16 belonged to Archaea (7 – *Halorcula*; 6 – *Halorubrum*; 1 – *Halobacterium*; 1 – *Halobaculum*; 1 – uncultured) while 1 belonged to Bacteria (*Idiomarina*). Rarefaction curves indicated that sediment samples harbored more OTUs than brine samples during both phases of salt production. The pattern of rarefaction curves further depicted that in most cases, the sampling depth was not sufficient to capture the entire diversity present in the solar saltern under investigation (Supplementary Figure S3). Chao1 and ACE values varied between 1095 to 2826 and 1175 to 3036, respectively. Based on the diversity indices and rarefaction curve patterns, the least species richness was observed for CP brine from ISH while EP sediment collected during the ISH phase displayed the highest species richness. Like rarefaction curves, Chao1 and ACE indices too indicated that sediments harbored a richer microbial community when compared to brine. Shannon diversity index values varied from 4.07 to 5.76 and the highest diversity was noticed among the sediment samples belonging to the ISH phase (Table 3). Dominance index varied from 0.016 to 0.074 in the brine samples and from 0.008 to 0.021 in the sediment samples during both phases of salt production. Buzas and Gibson’s evenness index varied from 0.103 to 0.239 for the brine samples and from 0.199 to 0.328 for the sediment samples during both phases of salt production. Dominance and Buzas and Gibson’s evenness indices implied that comparatively, the OTUs are distributed evenly and with equal dominance among sediments than brine. Similarities on the microbial community distribution between ISH and PSH phases were compared by ANOSIM and NMDS based on Bray-Curtis. NMDS plot indicated that there was no clear separation among the samples obtained indicating the similarity among the prokaryotic community composition between ISH and PSH which was further confirmed by ANOSIM (Figure 1). The statistical analysis revealed that there was no significant difference in the microbial community composition between brine (*R* = 0.03, *P* = 0.5) and sediments (*R* = 0.02, *P* = 0.6) obtained from both phases of salt production. Similarly, no significant difference was observed between brine and sediment samples obtained in ISH (*R* = 0.037, *P* = 0.6) and PSH (*R* = 0.307, *P* = 0.1).

**TABLE 2:** Bacterial and archaeal features obtained after processing the amplicon sequencing data through PANAM pipeline.

Saltern operation phase	Nature of the sample	Sampling sites	Total no of reads obtained	Total no of Bacterial reads	Total no of Archaeal reads	Total no of Bacterial OTUs	Total no of Archaeal OTUs
ISH	Brine	RP	402348	262738	139610	360	451
		EP	853366	419973	433393	291	519
		CP	502356	46682	455674	46	523
	Sediment	RP	5967	1021	4946	255	706
		EP	8997	1242	7755	170	915
		CP	4282	1716	2566	180	223
PSH	Brine	RP	589275	430437	158818	262	436
		EP	916698	172556	744142	34	602
		CP	862086	160566	701520	161	750
	Sediment	RP	14691	3746	10945	293	592
		EP	10320	3358	6962	371	588
		CP	6458	3285	3173	215	376

**TABLE 3:** Observed microbial richness and diversity estimates among the sampling sites of salterns of Siridao based on 97% OTU clusters.

Saltern operation phase	Nature of the sample	Sampling sites	OTUs observed	Shannon	Chao1	ACE^a^	Dominance	Buzas-Gibson’s Evenness
ISH	Brine	RP	810	5.26	1721	1818	0.017	0.239
		EP	809	5.23	2191	2291	0.016	0.231
		CP	568	4.07	1095	1175	0.074	0.103
	Sediment	RP	960	5.75	2221	2294	0.008	0.328
		EP	1083	5.76	2826	3036	0.010	0.294
		CP	983	5.70	2204	2231	0.011	0.306
PSH	Brine	RP	697	4.61	1795	1867	0.044	0.145
		EP	635	4.36	1555	1578	0.052	0.123
		CP	910	5.05	2267	2531	0.026	0.171
	Sediment	RP	884	5.48	2026	2060	0.013	0.272
		EP	958	5.54	2466	2552	0.012	0.266
		CP	590	4.76	1503	1633	0.021	0.199

*^*a*^ACE, Abundance-based coverage estimator. The diversity indices were calculated after normalizing the sequences obtained across all the sampling sites at 4100.*

### Dynamics of Archaea and Bacteria Along the Salinity Gradient

**FIGURE 2:**
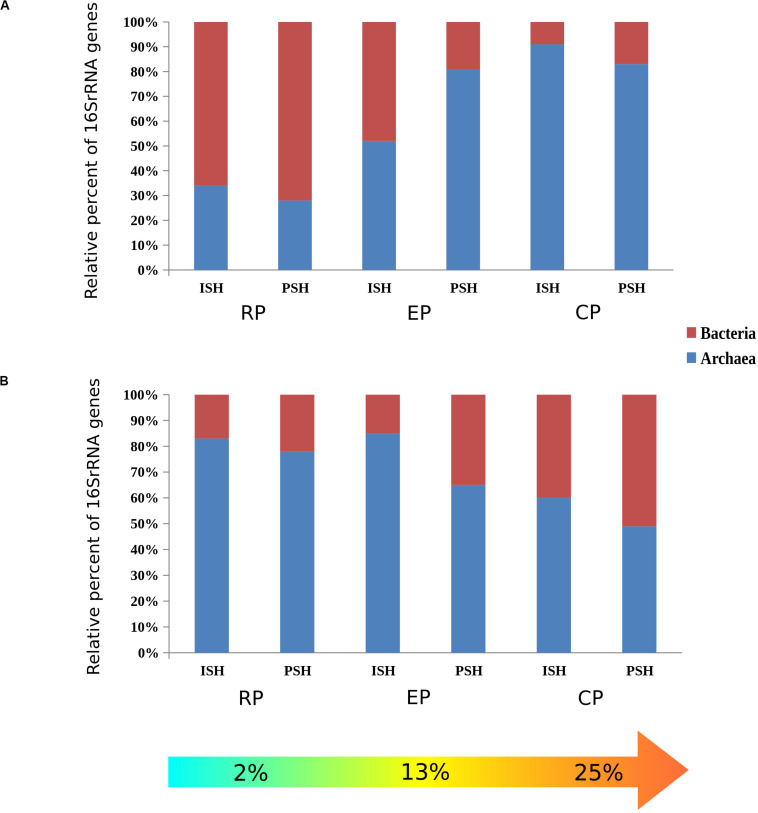
Horizontal stacked bar charts describing the relative abundance of Archaea and Bacteria between sampling sites with different salinities and between different salt producing season **(A)** Brine and **(B)** Sediment. ISH stands for initial salt harvesting phase, PSH for peak salt harvesting phase, RP for reservoir pan, EP for evaporator pan and CP for crystallizer pan.

The relative abundance of archaeal and bacterial phylotypes distributed across the salinity gradient during both phases of salt production was calculated based on the taxonomic affiliation of the sequences. Among brine samples, the occurence of archaeal sequences increased along the salinity gradient from 34% to 91% (ISH) and 27% to 84% (PSH) while bacterial sequences decreased from 66% to 9% (ISH) and 73% to 17% (PSH) (Figure 2A). However, in the sediment samples, an opposite trend was observed. Composition of bacterial sequences increased along the salinity gradient from 17% to 40% (ISH) and 21% to 51% (PSH) while archaeal sequences decreased from 83% to 60% (ISH) and 74% to 49% (PSH) (Figure 2B). This data further indicated that there was no difference in the relative frequency of archaeal and bacterial phylotypes between two salt-producing seasons. As a whole, during both the phases of salt production, along the increasing salinity gradient, archaeal sequences outnumbered bacteria proving that Archaea is the dominant prokaryotic member (Table 2).

### Community Composition of Archaea in Solar Salterns

**FIGURE 3:**
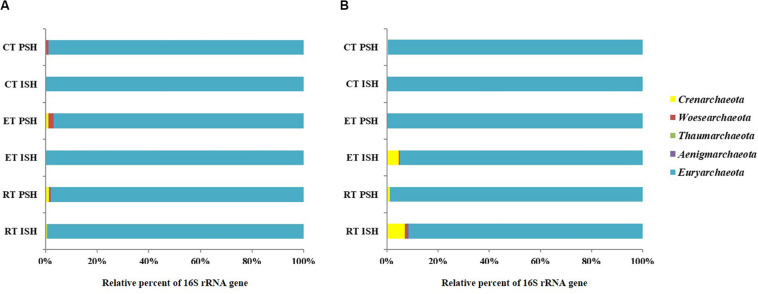
Relative sequence abundance at phyla level of the archaeal communities in **(A)** sediments and **(B)** brine of salterns of Siridao. ISH stands for initial salt harvesting phase, PSH for peak salt harvesting phase, RP for reservoir pan, EP for evaporator pan and CP for crystallizer pan.

**FIGURE 4:**
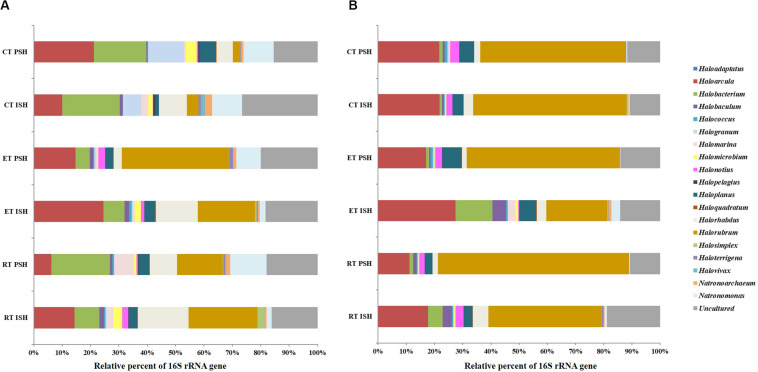
Relative sequence abundance at genera level of the archaeal communities in **(A)** sediments and **(B)** brine of salterns of Siridao. ISH stands for initial salt harvesting phase, PSH for peak salt harvesting phase, RP for reservoir pan, EP for evaporator pan and CP for crystallizer pan.

Archaeal sequences obtained from sediments were phylogenetically affiliated to five phyla namely *Crenarchaeota*, *Woesearchaeota*, *Aenigmarchaeota*, *Euryarchaeota*, and *Thaumarchaeota*, with *Euryarchaeota* being dominant and containing greater than 98% of the total sequences (Figure 3A). Sequences affiliated with *Crenarchaeota, Aenigmarchaeota*, and *Thaumarchaeota* were observed only in RP and EP. However, sequences belonging to *Woesearchaeota* were found across the salinity gradient from RP to CP albeit only during PSH. *Halobacteria* (including the predominant *Halobacteriaceae, Haloferaceae, Natrialbaceae*, and MSP41) was the dominant class of *Euryarchaeota* in sediment samples of Siridao salterns, contributing to 98.5% of the available sequences. The sequences were assigned to 19 halophilic archaeal genera. Further, major sequences at genus level (>1% among obtained sequences) across all salinities and during both phases of salt production in the sediments were identified as *Haloarcula*, *Halorubrum*, *Halorhabdus*, *Halobacterium, Natronomonas* and *Haloplanus* (Figure 4A). *Halorubrum* (24% in RP to 4% in CP and 15% in RP to 2% in CP during ISH and PSH respectively) and *Halorhabdus* (17% in RP to 9% in CP and 9% in RP to 5% in CP during ISH and PSH respectively) were found to decrease with increasing salinity during both phases of salt production. Sequences belonging to other genera like *Haloarcula* (14% in RP to 9% in CP and 8% in RP to 20% in CP during ISH and PSH respectively), *Halobacterium* (8% in RP to 20% in CP and 20% in RP to 18% in CP during ISH and PSH respectively), *Haloplanus* (2% in RP to 1% in CP and 3% in RP to 5% in CP during ISH and PSH respectively), *Natronomonas* (1% in RP to 10% in CP and 12% in RP to 10% in CP during ISH and PSH respectively) and *Halomicrobium* (3% in RP to 1% in CP and 1% in RP to 3% in CP during ISH and PSH respectively) were found to increase or decrease along the salinity gradient either during ISH or PSH (Supplementary Figure S4). Sequences affiliated with few genera were observed only at a particular salinity at specific sampling sites. For instance, *Halogranum* was observed exclusively in CP (6% and 12% during ISH and PSH respectively). Similarly, phylotypes associated with *Halomarina* was exclusively found to occur in the RP samples (2% and 6% during ISH and PSH respectively). Sequences affiliated with other genera like *Halobaculum*, *Halonotius*, and *Natronoarchaeum* were found to be sparsely occuring (between 1–2%) at intermittent sampling sites. About 15–26% of sequences across all compartments were assigned to uncultured haloarchaea (Figure 4A).

In brine, the obtained archaeal sequences were phylogenetically affiliated to four phyla namely *Crenarchaeota*, *Woesearchaeota*, *Aenigmarchaeota*, and *Euryarchaeota*, with *Euryarchaeota* being dominant, containing greater than 91% of the total sequences (Figure 3B). *Crenarchaeota* were found to occur only in RP (7%) and EP (4.7%) samples obtained during the ISH phase. Similar to sediments, *Halobacteria* (including predominant *Halobacteriaceae, Haloferaceae, Natrialbaceae*, and MSP41) was the dominant class of *Euryarchaeota* in brine samples of Siridao salterns, contributing to 99% available sequences. Further, the sequences were assigned to 17 halophilic archaeal genera. Major sequences at genus level (>1% of all obtained sequences) across all salinities and during both phases of salt production in the brine samples were identified as *Haloarcula*, *Halorubrum, Halonotius, Haloplanus* and *Halorhabdus* (Figure 4B). *Halorubrum* (39% in RP to 54% in CP and 67% in RP to 51% in CP during ISH and PSH respectively) and *Haloarcula* (17% in RP to 21% in CP and 11% in RP to 21% in CP during ISH and PSH respectively) were found to increase along the salinity gradient. Phylotypes affiliated with *Halobacterium* (5% in RP to 1% in CP) and *Halorhabdus* (5% in RP to 3% in CP) were found to decrease across the salinity gradient albeit in ISH phase while *Halonotius* (2% in RP to 3% in CP) and *Haloplanus* (3% in RP to 5% in CP) were found to increase along the salinity gradient in the PSH phase (Supplementary Figure S4). Similar to the sediment samples, sequences affiliated with *Halobaculum* were found to occur (between 1–4%) sparsely at intermittent sampling sites while about 10–18% of sequences were assigned to uncultured haloarchaea.

The cosmopolitan halophilic archaeal phylotypes, across the increasing salinity gradient and during both phases of salt production, were found to be *Haloarcula, Halorubrum*, and *Halobacterium*, often constituting more than 75% of the total sequences obtained. As a whole, the community composition of halophilic archaea was found to be relatively stable with a few archaeal phylotypes (*Haloarcula, Halorubrum, Halobacterium, Halorhabdus, Halogranum, Halomarina, Halobaculum, Haloplanus*, and *Natronomonas*) equally colonizing the salterns across different salinities.

### Community Composition of Bacteria in Solar Salterns

**FIGURE 5:**
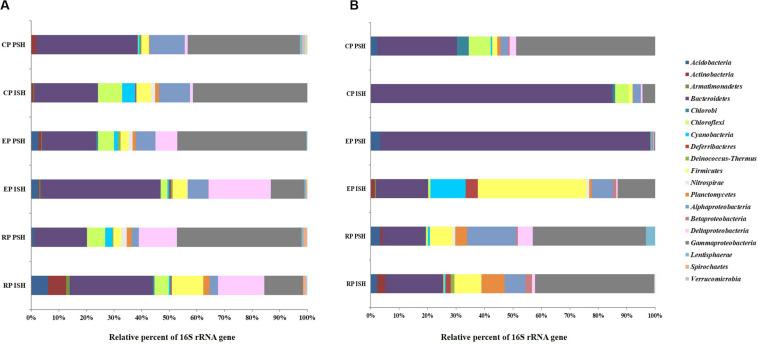
Relative sequence abundance at phyla level of the bacterial communities in **(A)** sediments and **(B)** brine of salterns of Siridao. ISH stands for initial salt harvesting phase, PSH for peak salt harvesting phase, RP for reservoir pan, EP for evaporator pan and CP for crystallizer pan.

Bacterial sequences obtained from sediment samples were taxonomically affiliated to 20 phyla (Figure 5A). The major sequences at phylum level (>1% of all obtained sequences), across all salinities and during both phases of salt production in the sediments were identified as *Bacteroidetes, Firmicutes* and *Proteobacteria*. Sequences belonging to certain phyla like *Chloroflexi* (5% in RP to 8% in CP and 6% in RP to 0.2% in CP during ISH and PSH, respectively) were found to increase along the salinity gradient during ISH and vice versa during PSH. Relative sequence frequency of *Firmicutes* (11% in RP to 2% in CP) was found to be high along the increasing salinity gradient albeit exclusively during ISH phase. Sequences belonging to *Acidobacteria* (6% in RP and 2% in EP during ISH and PSH respectively) and *Actinobacteria* (6% in RP during ISH) were found to be abundant in RP and EP. Sequences affiliated with phylum *Proteobacteria*, class *Gammaproteobacteria* (13% in RP to 41% in CP and 45% in RP to 40% in CP during ISH and PSH respectively) and *Alphaproteobacteria* (2% in RP to 11% in CP and 2% in RP to 12% in CP during ISH and PSH respectively) increased while *Deltaproteobacteria* (16% in RP to 1% in CP and 13% in RP to 1% in CP during ISH and PSH respectively) decreased along the increasing salinity gradient during both phases of salt production (Supplementary Figure S4). Unlike Archaea, there were no dominant bacterial phylotypes across salinity gradient and throughout the salt production process (Table 4). Phylotypes belonging to *Bacillus* (except CP PSH) and *Halomonas* (except RP PSH) were found frequently across all sampling locations while *Rubricoccus* was observed consistently across the salinity gradient during ISH phase. In contrast, phylotypes affiliated to *Thioalkalispira* and *Desulfovermiculus* were observed in RP and EP while *Marinobacter* and *Marinicella* were observed in EP and RP sediment samples, across both phases of salt production. Apart from the culturable representatives, we could also observe sequences with similarity to the uncultured *Bacteroidetes* clone, ML602J-37 (1.6% in RP to 14.4% in CP and 1.5% in RP to 19% in CP during ISH and PSH respectively) along the salinity gradient. Similarly, sequences affiliated to uncultured *Chitinophagaceae* were observed to increase along salinity gradient during ISH (18.6% in RP to 28.9% in EP) and PSH (10.8% in RP to 15% in CP) phases. Further, we could affiliate about 14.3% sequences obtained from CP PSH to Uncultured *Ectothiorhodospiraceae*

**TABLE 4:** Bacterial diversity at the genus level among the various sampling sites of salterns of Siridao.

Sampling Sites	Sediment		Brine	
	Genus	Relative abundance in percent	Genus	Relative abundance in percent
ISH – RP	Uncultured *Chitinophagaceae*	18.6%	*Halomonas*	25.8%
	*Delsulfovermiculus*	12.9%	*Planctomyces*	4.8%
	*Rubricoccus*	5.6%	Uncultured *Clostridiaceae*	4.3%
	Uncultured *Xanthomonadales*	5.1%	*Fulvivirga*	3%
	*Bacillus*	3.9%	*Gracilimonas*	3%
	*Acidothermus*	2.7%	ML602J-37	3%
	*Dialister*	2.3%	*Salegentibacter*	2.5%
	Uncultured *Acidobacteriaceae*	2.1%	*Marinicella*	2.2%
	Uncultured *Anaerolineaceae*	2.1%	*Alcanivorax*	1.8%
	*Thioalkalispira*	1.8%	Uncultured *Deferribacterales*	1.8%
	*Halomonas*	1.6%	Uncultured *Xanthomonadaceae*	1.5%
	ML602J-37	1.6%	*Idiomarina*	1.6%
			*Truepera*	1.2%
			*Methylophaga*	1.1%
ISH – EP	Uncultured *Chitinophagaceae*	28.9%	*Bacillus*	21.6%
	*Desulfovermiculus*	18.8%	*Halomonas*	7.9%
	*Rubricoccus*	5.6%	*Psychroflexus*	7.7%
	ML602J-37	4.3%	*Cladithrix*	4.1%
	*Rhodovulum*	3.2%	ML602J-37	3.8%
	*Bacillus*	2.3%	Uncultured *Saprospiraceae*	3.2%
	*Thiohalorhabdus*	1.9%	*Lysinibacillus*	1.8%
	*Halomonas*	1%	Uncultured *Paenibacillaceae*	1.8%
			*Tropicimonas*	1.6%
			*Marinobacter*	1.6%
			*Phaeobacter*	1.2%
			*Rathybacter*	1.1%
			*Ruegaria*	1%
ISH – CP	ML602J-37	16.7%	*Rubricoccus*	79%
	*Halomonas*	12.3%	Uncultured *Chitinophagaceae*	3%
	*Marinobacter*	11.2%	ML602J-37	2.7%
	Uncultured *Anaerolineaceae*	5.6%	*Halomonas*	2.2%
	*Bacillus*	3.4%		
	Uncultured *Saprospiraceae*	3.2%		
	*Sediminimonas*	3.1%		
	Uncultured *Caldilineaceae*	2.6%		
	Uncultured *Rhodobacteraceae*	2%		
	*Marinicella*	1.7%		
	*Rubricoccus*	1.2%		
PSH – RP	*Thioalkalispira*	15.3%	*Acidoferrobacter*	13.6%
	Uncultured *Chitinophagaceae*	10.8%	Uncultured *Clostridiaceae*	6.1%
	Uncultured *Anaerolineaceae*	5.8%	*Magnetospira*	5%
	*Delsulfovermiculus*	3.3%	*Idiomarina*	4.5%
	*Desulfosalsimonas*	2.3%	*Halomonas*	4%
	Uncultured *Gemmatimonadaceae*	2%	*Fulvivirga*	3.9%
	Uncultured *Marinilabiaceae*	2%	*Gramella*	3.9%
	*Robiginitalea*	2%	Uncultured *Victivallaceae*	3.2%
	*Bacillus*	1.5%	*Planctomyces*	2.3%
	Uncultured *Desulfobacteraceae*	1.5%	*Acanthopleuribacter*	1.9%
	ML602J-37	1.5%	*Thiohalophilus*	1.6%
PSH – EP	*Halovibrio*	11.8%	*Rubricoccus*	94.3%
	*Thioalkalispira*	10.1%	Uncultured *Acidobacteriaceae*	3.2%
	*Marinobacter*	7.1%		
	ML602J-37	5.6%		
	*Rubricoccus*	5.3%		
	*Robiginitalea*	2.8%		
	Uncultured *Anaerolineaceae*	2.7%		
	Uncultured *Chitinophagaceae*	2.5%		
	*Halomonas*	2.2%		
	Uncultured *Caldilineaceae*	1.8%		
	*Marinicella*	1.4%		
	Uncultured *Desulfobacteraceae*	1.3%		
	*Bacillus*	1.2%		
	Uncultured *Gemmatimonadaceae*	1.2%		
	*Desulfosalsimonas*	1.1%		
	Uncultured *Acidobacteriaceae*	1%		
PSH – CP	ML602J-37	19.6%	*Halovibrio*	31.7%
	Uncultured *Chitinophagaceae*	15%	*Rubricoccus*	17.3%
	Uncultured *Ectothiorhodospiraceae*	14.3%	Uncultured *Chitinophagaceae*	5.5%
	*Marinobacter*	10.5%	Uncultured *Caldilineaceae*	3.1%
	*Ruegeria*	4.8%	Uncultured *Acidobacteriaceae*	2%
	*Albidovulum*	1.8%	Uncultured *Anaerolineaceae*	1.8%
	*Marinicella*	1.4%	ML602J-37	1.7%
	*Halomonas*	1.3%		
	*Rathybacter*	1.1%		

*16S rRNA gene sequences greater than 1% relative abundance alone were considered.*

Bacterial sequences obtained from brine samples were taxonomically affiliated to 17 phyla (Figure 5B). The major sequences at phylum level (>1% of all obtained sequences) across all salinities and during both phases of salt production in the brine were identified as *Bacteroidetes* and *Proteobacteria*. Sequences belonging to *Firmicute*s (9% in RP to 1% in CP and 7% in RP to 1% in CP during ISH and PSH respectively) and *Proteobacteria* (52.5% in RP to 7.9% in CP and 62.5% in RP to 49.9% in CP during ISH and PSH respectively) were found to decrease while *Bacteroidetes* (20% in RP to 85% in CP and 15% in RP to 25% in CP during ISH and PSH respectively) was found to increase along a salinity gradient during both phases of salt production. Sequences affiliated to *Planctomycetes* (4% in RP to 1% in CP) and *Acidobacteria* (3% in RP to 2% in CP) were found to decrease along the salinity gradient during the PSH phase. *Actinobacteria* (2% in RP-ISH), *Cyanobacteria* (12% in EP-ISH) and *Deferribacteres* (4% in EP-ISH) were found to be abundant at intermittent sampling sites. Within the phylum *Proteobacteria*, *Gammaproteobacteria* (41% in RP to 4% in CP and 39% in RP to 44% in CP during ISH and PSH respectively) were found to decrease along the salinity gradient in the ISH phase and vice versa during PSH phase. *Alphaproteobacteria* (7% in RP to 2% in CP and 17% in RP to 2% in CP during ISH and PSH respectively) was found to decrease along the salinity gradient during both phases of salt production while the same was observed for *Deltaproteobacteria* (5% in RP to 2% in CP), however only during PSH (Supplementary Figure S4). Like sediment samples, there were no common phylotypes (>1% of all obtained sequences) observed across all the sampling sites. Sequences belonging to *Halomonas* (25.8% in RP to 2.2% in CP) decreased along the salinity gradient in the ISH phase while *Rubricoccus* was particularly dominant in the EP (94.3%) and CP (79% in ISH and 17.3% in PSH) during both phases of salt production (Table 4). Sequences affiliated to *Bacillus* and *Halovibrio* were observed majorly at defined salinities and during a particular phase of salt production. For example, *Bacillus* was dominant (21.6%) in EP PSH brine while *Halovibrio* was dominant (31.7%) in CP PSH brine. However, sequences belonging to *Idiomarina*, *Planctomyces*, and *Fulvivirga* were confined exclusively to RP brine during both phases of salt production. Unlike sediment samples, sequences of ML602J-37 (3% in RP and 2% in CP) in brine were found to decrease along the salinity gradient in the ISH phase (Table 4). Similarly, sequences belonging to uncultured *Chitinophagaceae* was observed in CP (3% and 5.5% in ISH and PSH) samples while Uncultured *Clostridiaceae* was observed in RP (4.3% and 6.3% in ISH and PSH) samples.

Based on the taxonomic classification of reads obtained, *Bacteroidetes* and *Proteobacteria* were the dominant bacterial phyla distributed across the salinity gradient and omnipresent during both phases of salt production. Overall, sediments exhibited a higher diversity compared to brine indicated through the presence of sequences belonging to higher number of phyla and a correspondingly greater number of bacterial genera. Though *Halomonas*, *Rubricoccus*, and *Bacillus* were the dominant bacterial phylotypes in the solar saltern under study, they could not be observed at all sampling sites. Similarly, phylotypes belonging to *Marinobacter* and *Marinicella* were exclusive to sediment samples while *Halovibrio* was exclusive to brine.

## Discussion

Scant information is available regarding the microbial diversity of highly transient hypersaline environments like the salterns of Siridao, Goa. In this study, the prokaryotic community composition of a solar saltern has been profiled over a salinity gradient using a metabarcoding approach employing high-throughput sequencing technology.

### Coastal Salterns of Siridao Are One of the Most Diverse Solar Salterns Around the World

In this study, we obtained about 10-fold higher reads in brine samples compared to sediments. Much lower read numbers from sediment samples could be attributed to the difficulty in the extraction of nucleic acids, despite trying several commercial kits and in-house DNA extraction protocols, the quality of DNA obtained was relatively inferior compared to brine samples. These sediments were predominantly composed of clay and therefore were recalcitrant to DNA extraction procedures. This difficulty was further compounded by the high salinity of the sediment samples (Cai et al., 2006). Despite the low read numbers, the number of OTUs obtained in the sediments were comparable to similar studies carried out at salt marshes in Odiel estuary, Spain (Vera-Gargallo et al., 2019) and a solar saltern located at West Bengal, India (Sen and Mukhopadhyay, 2019) focusing on the microbial diversity of hypersaline soils.

The diversity indices obtained from the salterns of Goa were comparable against the diversity of similar thalassohaline ecosystems like solar salterns located in USA (Youssef et al., 2012), India (Sen and Mukhopadhyay, 2019), Turkey (Çinar and Mutlu, 2016), and Spain (Gómez-Villegas et al., 2018), soda ash concentration ponds in Ethiopia (Simachew et al., 2016) and graduation towers from Poland (Kalwasińska et al., 2018). When the diversity between the two salt production phases was compared, samples from the ISH phase indicated an overall higher diversity owing to the moderately halophilic and halotolerant regime prevailing before the start of the salt production process. However, a sustained high salinity during the PSH restricted the growth of non-halophilic microorganisms, otherwise prevalent in ISH, resulting in the lower diversity values. In general, sediments exhibited a greater diversity in terms of OTU to sequence ratio compared to brine. This is in support of the view that sediments support a broad range of microorganisms because of the availability of a wide range of organic nutrients and hypersaline soils are no exception to this pattern (Sander and Kalff, 1993; Kimbrel et al., 2018). Though the statistical analysis indicated that the taxonomic composition between the brine and sediments during different salt production phases are similar, we acknowledge the low statistical power involved in the analysis due to the composite sampling approach resulting in single sample per sampling location.

High diversity prevailing in these ecosystems could be owed to the stable tropical climate and high annual precipitation resulting in the introduction of diverse microbes (Peter et al., 2014; Couto-Rodríguez and Montalvo-Rodríguez, 2019). Salterns of Siridao receive annual rainfall in the range of 280–480 cm primarily from the beginning of June till November, flooding the salterns and this event provides an opportunity for the introduction of non-halophilic microbes through runoffs from adjacent environments and urban dwellings. Further, this is in accordance with a study carried out by Youssef et al. (2012), indicating that hypersaline environments that are in constant salinity fluctuations tend to possess a diverse non-halophilic, halotolerant and extremely halophilic members.

### Archaea Dominates the Low and Moderate Salinity Sediments

It is commonly observed that there is an increase in archaeal phylotypes in brine along the increasing salinity gradient with a simultaneous decrease in Bacteria (Benlloch et al., 2002; Casamayor et al., 2002; Baati et al., 2008; Ghai et al., 2011). Though our results corroborated with the established trend in brine, we could observe Archaea being dominant over Bacteria in sediments too, during both phases of salt production. However, microbial diversity studies in hypersaline soils and sediments suggest that there is a dominance of Bacteria, especially among the samples obtained from low and moderate salinities (Sørensen et al., 2005; Pandit et al., 2015). Further, the studies carried out by Robertson et al. (2009) on the hypersaline microbial mats at Guerrero Negro solar salterns, Wang et al. (2007) on the saline sediments obtained from the solar salterns of Taiwan, and Vera-Gargallo et al. (2019) on the hypersaline sediments at the Odiel salt marshes of Spain have estimated that Bacteria constituted about 90, 53, and 78% among the total prokaryotes respectively. Contrary to the existing literature, bacterial phylotypes were outnumbered by archaea in sediments of solar salterns of Siridao. With the onset of salinity, Archaea could possibly compete with Bacteria and colonize the saltern compartments at a faster rate. Once the prokaryotic community structure is established with Archaea being the dominant prokaryote, the structure remains less perturbed during the continued operation of the saltern (Barin et al., 2015).

One of the most remarkable findings of our study is the prevalence of archaeal sequences among the RP sediment samples. Though the occurrence of halophilic archaea in the low salinity environments has been documented before, the large number of sequences observed in the salterns of Siridao is unprecedented. Recently, Kimbrel et al. (2018) have shown that at 7.5% salinity, there were about 20% and 18% of archaeal sequences in brine and sediments obtained from a solar saltern respectively. Similarly, Elshahed et al. (2004) have reported the isolation of halophilic archaeal clones belonging to *Halogeometricum*, *Natronomonas*, *Halococcus*, and *Haloferax* from low salinity (0.7 – 1%) brine and sediment samples. Walsh et al. (2005) have also reported the isolation of archaeal clones belonging to *Euryarchaeota* from low salinity sediments. Purdy et al., 2004 have reported the isolation of *Haloferax* and *Halogeometricum* from low salinity estuary sediments while Braganca and Furtado (2009) have reported the isolation of *Haloarcula* and *Halobacterium* from various low salinity eco-niches in close vicinity to the study site. One possible reason for the survival of halophilic archaea in the low salinity samples of Siridao salterns could be attributed to the predominant red clay in saltern sediments (Fukushima et al., 2007). These clay particles could act as micro-niches for halophilic archaea because they contain micropores which eventually are filled up by brine along with the microbes. Further, provided that the clay particles are negatively charged and could hold up cations like Na^+^ and K^+^, establishing a conducive environment for the survival of halophilic archaea even during unfavorable conditions (Rožić et al., 2000). Despite their gene abundance at low salinities, the viability of halophilic archaea remains questionable and should be assessed by other molecular techniques.

Another interesting finding of the study is the high sequence percentage of Archaea prevalent at the intermediate salinities. Studies carried out by Ghai et al. (2011) on a 19% salinity brine sample and Fernandez et al. (2014a) on a 13% salinity brine sample have shown that about 46% and 27% of the total 16S rRNA gene sequences could be attributed to Archaea respectively. This indicates that the Archaea plays a significant part in the community composition of prokaryotes at intermediate salinities. In our study, we could also detect a significant proportion of Archaea at intermediate salinities albeit at a higher rate than previously reported ranging between 55% and 85% in brine and sediments, respectively. As the saltern compartments are interconnected and the prevalent Archaea in RP could provide a microbial seed bank, microorganisms could be transported from RP to CP via EP through a constant influx of brine (Lennon and Jones, 2011; Kimbrel et al., 2018). This could provide Archaea a head start over Bacteria to rapidly colonize and dominate in EP and CP.

### Halophilic Archaea Tolerate Wide Salinity Fluctuations and Show Minimal Dependency on Magnesium

*Halorubrum*, the most diverse cultivable halophilic archaeal genus containing about 39 culturable representatives (Parte, 2018) and reported to be a constituent member of solar salterns and hypersaline environments around the world (Ochsenreiter et al., 2002; Burns et al., 2004; Baati et al., 2008; Çinar and Mutlu, 2016; Kambourova et al., 2016) was very abundant genus in the salterns of Siridao too. Given its low dependency on magnesium (0.005–0.6 M) and a wide range of NaCl tolerance (1.0–5.2 M) (Oren, 2018), *Halorubrum* appears to be the most suitable to colonize the highly dynamic Siridao salterns. *Haloarcula*, though not frequently reported to be encountered in metagenomic studies (Ochsenreiter et al., 2002; Dillon et al., 2013; Plominsky et al., 2014; Çinar and Mutlu, 2016) was seen in the salterns of Siridao. Their incidence could be attributed to the ability to metabolize a wide range of substrates and possessing very similar characteristics to *Halorubrum* in terms of magnesium (0.005–0.6 M) and NaCl (1.7–5.2 M) dependency (Ventosa et al., 2019). One of the interesting findings of the study is the occurence of *Halobacterium* in Siridao salterns, especially in sediments. Despite, *Halobacterium* being one of the earliest described halophilic archaeal genus and isolated from various hypersaline environments like salterns, salt lakes, salted fish and salted hides, it has been scarcely found in metagenomic studies (Maturrano et al., 2006; Zafrilla et al., 2010; Baati et al., 2011; Plominsky et al., 2014). Since *Halobacterium* possess higher NaCl (2.5–5.2 M) and magnesium (0.05–0.6 M) requirements coupled with versatile anaerobic respiration, CP sediments appear to be a suitable habitat for their prevalence (Oren and Ventosa, 2017). Consistent with previous studies, *Natronomonas*, an extremely halophilic archaeon (requiring at least 2 M NaCl) was found in CP sediment. Though *Natronomonas* has been frequently correlated with halophilic alkaine environments, a few species have been recovered from neutrophilic environments as well. Therefore, sequences affiliated to *Natronomonas* recovered from Siridao saltern could possibly be neutrophilic, low-magnesium dependent phylotypes (Minegishi and Kamekura, 2015). Other genera found in this study, *Halorhabdus* (Mouné et al., 2003; Baati et al., 2011; Makhdoumi-Kakhki et al., 2012; Baricz et al., 2014) and *Haloplanus* (Baricz et al., 2014; Vera-Gargallo and Ventosa, 2018; Couto-Rodríguez and Montalvo-Rodríguez, 2019) are the common dwellers of solar salterns around the world while *Halogranum* (Youssef et al., 2012) and *Halomicrobium* (Youssef et al., 2012; Castelán-Sánchez et al., 2019) have been rarely documented. Interestingly, almost all of the halophilic archaea reported in Siridao salterns have been recorded to possess wide NaCl tolerance (0–5.2 M) with minimal or no dependency on magnesium.

The prevalence of *Haloquadratum*, a cosmopolitan halophilic archaeal genus in the crystallizer pans around the world (Maturrano et al., 2006; Baati et al., 2011; Ghai et al., 2011; Dillon et al., 2013) was found to be negligible (<0.3% of available reads) in Siridao salterns. Few studies have also reported the low or negligible occurrence of *Haloquadratum* in salterns that are very similar in operation with Siridao salterns and other hypersaline environments (Pašić et al., 2005; Wang et al., 2007; Manikandan et al., 2009; Kalwasińska et al., 2018). This may be explained by the lower level of magnesium prevalent in the saltern samples. Solar salterns of Goa are characterized by low retention time resulting in the brine replenishment daily and therefore the concentration of magnesium remains tightly regulated. This is in contrast to the well-studied Bras del Port saltern in Spain and Cabo Rojo salterns in Puerto Rico, where the brine is replenished every year and once in two months respectively (Pašić et al., 2014; Couto-Rodríguez and Montalvo-Rodríguez, 2019). Further, a study carried out by Podell et al. (2014) has shown that *Haloquadratum* displayed a positive correlation with elevated magnesium concentration and negative correlation with *Halorubrum* and *Haloarcula*. Therefore, our study indicates that low retention time, low magnesium concentration, and competition from other fast-growing halophilic archaea for space prevent *Haloquadratum* from becoming a dominant halophilic archaeal genus. The low retention time of brine could also be attributed to the relatively low incidence of *Halonotius*, which requires at least 2.7 M NaCl for growth (Oren, 2016) and uncultured Nanohaloarchaeota, frequently encountered in hypersaline environments (Narasingarao et al., 2012; Finstad et al., 2017; Mora-Ruiz et al., 2018). Another possibility of the absence of Nanohaloarchaeota could be owed upon the absence of a specific host because these organisms have been found to dwell as symbionts with other halophilic archaea (Hamm et al., 2019). A previous study carried out in Ribandar salterns employing culture-dependent techniques, located at a distance of 10 km from the site under study, reported the isolation of members belonging to *Halococcus* during ISH phase and *Halorubrum*, *Halococcus*, *Haloferax*, and *Haloarcula* during PSH phase (Mani et al., 2012a). However, in Siridao salterns, *Halococcus* constituted a minor fraction (<1%) and *Haloferax* was not encountered in any of the samples studied. This shows the variation in the halophilic archaeal community structure between the closely located solar salterns despite similar operational procedures and geophysical conditions.

### Major Shifts in Bacterial Diversity Between Salt Production Phases and Compartments

Like several other salterns and hypersaline environments, Siridao salterns support diverse communities of phylotypes belonging to *Bacteroidetes* and *Gammaproteobacteria* (Sørensen et al., 2005; Baati et al., 2011; Zhang et al., 2016). The occurence of *Alphaproteobacteria*, *Betaproteobacteria* and *Firmicutes* decreased with increasing salinity which was consistent with the results obtained by Oueriaghli et al. (2018). A study carried out in the same salterns under investigation, employing DGGE had recovered sequences affiliated with *Gammaproteobacteria*, supporting their importance at Siridao salterns (Mani et al., 2015).

Interestingly, *Rubricoccus*, one of the dominant bacterial genera in the Siridao salterns, has a growth range between 1–5% NaCl (w/v) and was originally isolated from seawater (Park et al., 2011). However, this particular bacterium has seldom been found in hypersaline environments and not been reported in any metagenomic studies to the best of our knowledge. The prevalence of this genus in EP and CP could indicate novel phylotypes related to *Rubricoccus* dwelling in salterns of Siridao that are yet to be explored. Another abundant genus like *Halomonas* is a versatile moderately halophilic bacterium with a very wide salinity range [0–5.2 M NaCl (w/v)] (Vreeland, 2015) and are commonly encountered in salterns (Tsiamis et al., 2008; Boujelben et al., 2014; Oueriaghli et al., 2014). Sediments showed the occurrence of *Marinobacter*, the denitrifying bacterium *Halovibrio*, sulfate-reducing anaerobic bacterium *Desulfovermiculus* and sulfur-oxidizing microaerophilic bacterium *Thioalkalispira*. As Siridao salterns are in the vicinity of urban dwellings and industries, hydrocarbons and organic acids may eventually get concentrated in the salterns. This would provide an opportunity for the moderately halophilic and facultative anaerobic bacteria capable of degrading hydrocarbon like *Marinobacter* (Handley and Lloyd, 2013; Bowman and McMeekin, 2015) and *Halovibrio* (Sorokin et al., 2006) to flourish in EP and CP sediments. Though the presence of halophilic *Desulfovermiculus* in hypersaline environments has been recorded before (Roychoudhury et al., 2013; McGonigle et al., 2019), the incidence of *Thioalkalispira*, originally isolated from alkaline soda lake sediments and with a pH range of 8.4 -10 is truly surprising (Sorokin, 2008; Sorokin et al., 2013). Together with *Desulfovermiculus* and *Thioalkalispira*, the presence of sulfur-oxidizing *Acidoferrobacter* in RP indicates the active sulfur metabolism occurring in the RP and EP sediments. *Bacillus* has been frequently reported from the solar salterns of India (Kumar et al., 2015; Suganthi et al., 2018; Sen and Mukhopadhyay, 2019). Given the dynamic nature of the solar salterns of India being operated and halotolerance of *Bacillus* in surviving salinities up to 25% NaCl (w/v) (Garabito et al., 1998), this genus could be dominating the salterns during the non-operational phases. In the case of Siridao salterns, their diminishment could be due to the sustained salinity during PSH, paving the way for moderately and extremely halophilic bacteria to colonize the salterns.

At majority of the sampling sites, uncultured phylotypes belonging to phylum *Bacteroidetes* were found to be abundant, sometimes outnumbering their culturable counterparts. Interestingly, uncultured *Bacteroidetes* clone ML602J-37, initially recovered from alkaline Mono lake in USA (Humayoun et al., 2003), was abundant in EP and CP sediments. Though it has been recovered from other neutral environments like soil samples from Antarctica (Shravage et al., 2007), stromatolites from Argentina (Toneatti et al., 2017), and Keke Salt Lake from China (Han et al., 2017), the presence of ML602J-37 in solar saltern samples requires a detailed investigation of their role in the metabolic activity of sediments. Similarly, abundance of uncultured *Chitinophagaceae* in sediments indicates novel phylotypes thriving in salterns of Siridao that are yet to be explored.

*Salinibacter* (Antón et al., 2008; Baati et al., 2008; Di Meglio et al., 2016) and *Spiribacter* (Fernandez et al., 2014a, b; León et al., 2014, 2017), most widely represented bacterial genera at moderate and high salinities across global hypersaline environments failed to be identified in the Siridao salterns. This could be attributed to the relatively low salinities and transient nature of the Siridao salterns since the occurence of *Salinibacter* has been shown to strongly correlate with increasing and sustained salinity (Pandit et al., 2015). On the other hand, though *Spiribacter* could tolerate wide salinity fluctuations (0-2 M NaCl), its growth peaks at very specific salinity (0.8 M NaCl) making it difficult to flourish in highly fluctuating salinities (León et al., 2018). Few studies carried out in the salterns of Chile, Argentina, Poland, and Spain have also reported the absence or low incidence of *Salinibacter* (Kalwasińska et al., 2018; Mora-Ruiz et al., 2018; Oueriaghli et al., 2018). Further, considering the absence of dominant archaeal phylotype of *Haloquadratum* and bacterial phylotypes such as *Salinibacter* and *Spiribacter*, we are in complete agreement with the views drawn by Gomariz et al. (2015) that solar salterns, despite being called ‘steady-state’ systems, undergo various microbial fluctuations at similar salinities and therefore general conclusions should be drawn with caution.

## Conclusion

Artisanal solar salterns of Goa are of rich historical and cultural importance. Their transient nature makes these salterns unique ecosystems for studying the effect of salinity fluctuations on the prokaryotic community structure. On analyzing the microbial community structure between two salt production seasons, indicated the prevalence of a similar prokaryotic composition. Further, this study has clearly shown that Archaea was stable and dominant despite encountering wide changes in salinity between two different salt production phases, while significant fluctuations were observed in bacterial community structure. Among the archaeal phylotypes, *Halorubrum* and *Haloarcula* were dominant while *Rubricoccus* and *Halomonas* were the dominant bacterial phylotypes. Thus, the prokaryotic community structure of salterns of Siridao is mainly driven by halophilic archaea and bacteria that can withstand wide fluctuations in salinity and have a low dependency on ionic constituents. A thorough assessment of these ecosystems through metagenomic approaches can help us in understanding the adaptations and functioning of the microbiome in transient conditions.

## Data Availability Statement

The raw sequencing data generated in this study have been deposited in the SRA under BioProject PRJNA614987.

## Author Contributions

JB, GB, and DD conceived the study. KM and JB conducted sampling. KM and MH performed DNA extraction, PCR and library preparation for Illumina sequencing. KM, NT, and GB carried out the data analysis and statistical calculations. KM, MH, and NT drafted the manuscript. All authors read and approved the final manuscript.

## Conflict of Interest

The authors declare that the research was conducted in the absence of any commercial or financial relationships that could be construed as a potential conflict of interest.

## Footnotes

1 https://github.com/panammeb/stable
