# Supplementary material for: Transient Dynamics of Archaea and Bacteria in Sediments and Brine Across a Salinity Gradient in a Solar Saltern of Goa, India
Source: Front Microbiol. 2020 Aug 13;11:1891. doi: 10.3389/fmicb.2020.01891 (PMC7461921; doi:10.3389/fmicb.2020.01891)
Supplement: Supplementary file 1 [file Data_Sheet_1.PDF]

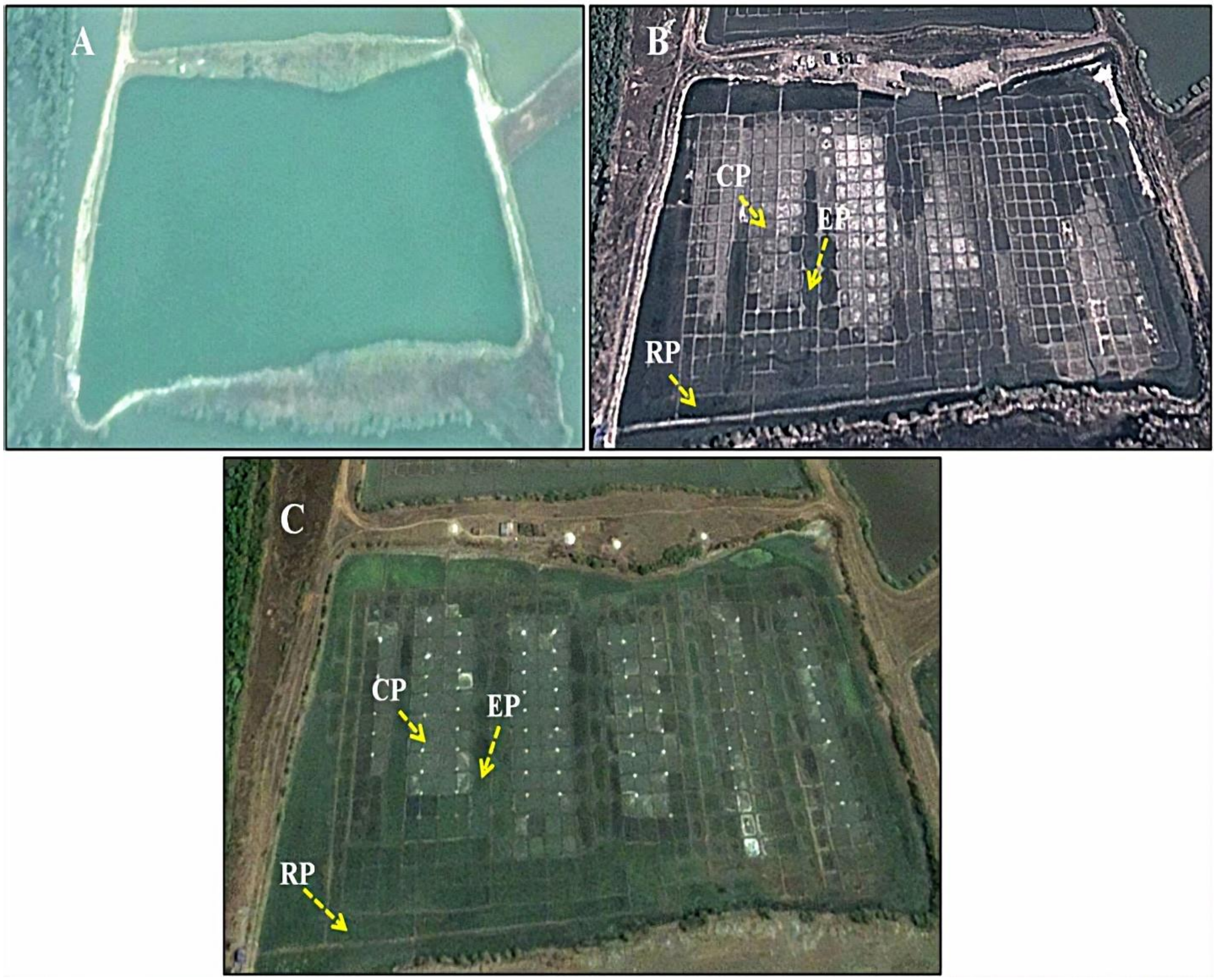

Supplementary Figure 1: Map showing the organizational structure of a coastal solar saltern located at Siridao, Goa, India A) Salterns flooded with rain water during monsoon (October 2013); B) Salterns during the initial salt harvesting (ISH) phase (February 2014) and C) Salterns during peak salt harvesting (PSH) phase (May 2014). Reservoir pans are usually thrice in size compared to evaporator or crystallizer pans, to facilitate water storage during high tides. The salt crystals collected at the interjection between two crystallizer tanks are observed as white dots. Reservoir pan (RP), evaporator pan (EP) and crystallizer pan (CP) indicate the location of sampling sites.

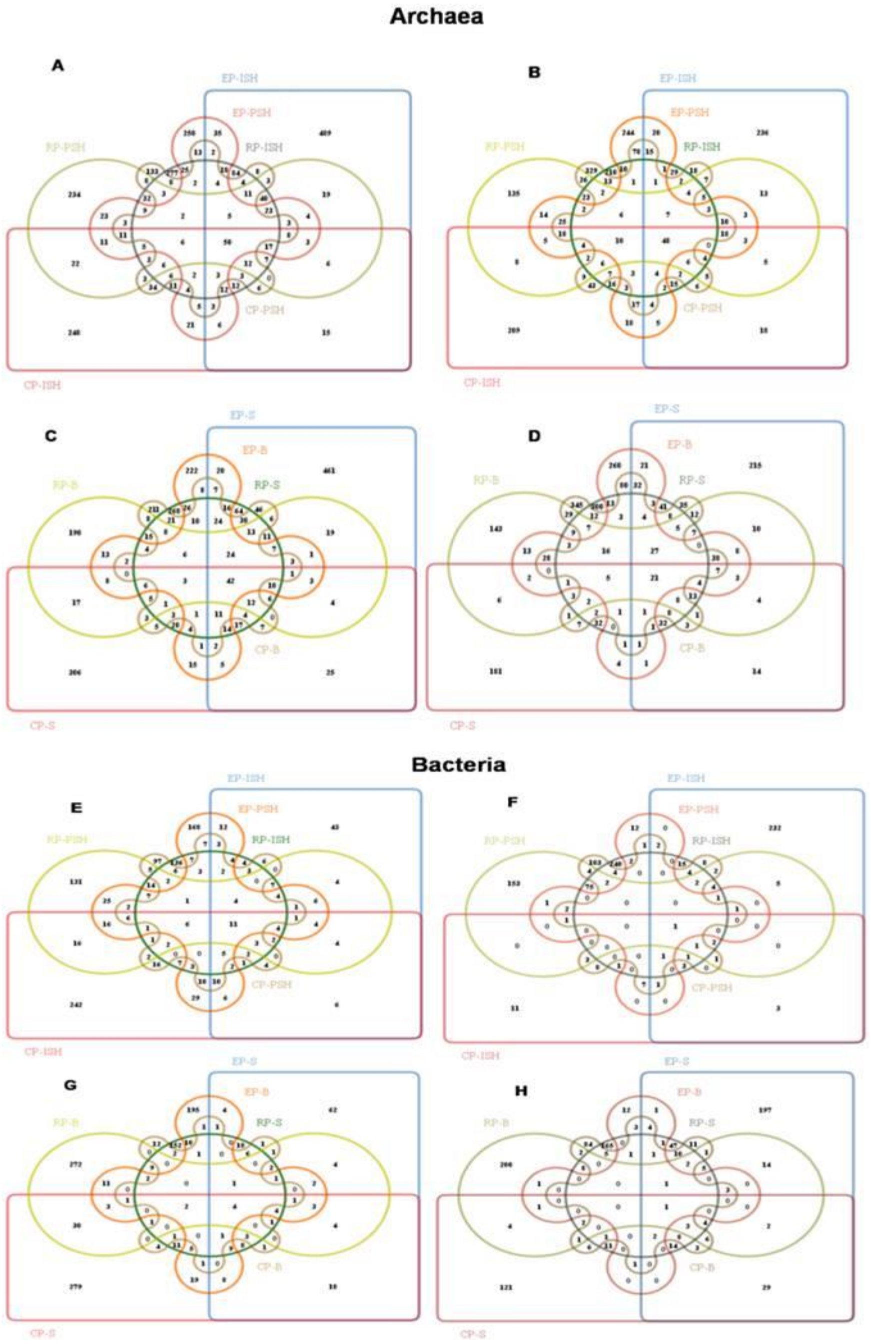

Supplementary Figure 2: Venn plot showing the common archaeal OTUs distributed between **(A)** sediment samples from ISH and PSH, **(B)** brine samples from ISH and PSH, **(C)** Sediment and brine samples from ISH, **(D)** Sediment and brine samples from PSH. Common bacterial OTUs distributed between **(E)** sediment samples from ISH and PSH, **(F)** brine samples from ISH and PSH, **(G)** Sediment and brine samples from ISH, **(H)** Sediment and brine samples from SH.

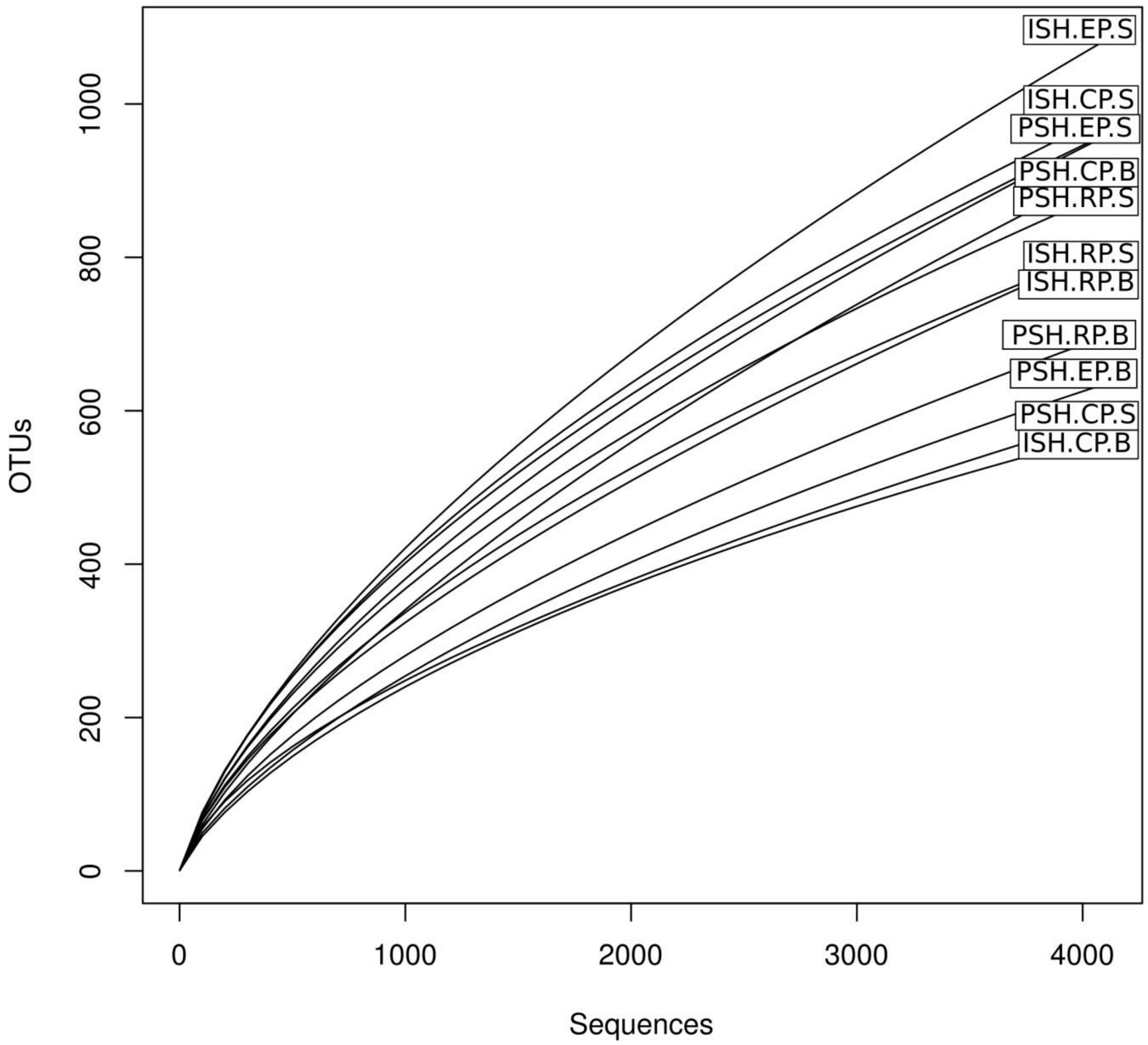

Supplementary Figure 3: Rarefaction curves depicting the OTU richness between the various samples. Curves were plotted after rarefying to 4100 sequences. ISH - initial salt harvesting phase, PSH - peak salt harvesting phase, RP - Reservoir pan, EP - evaporator pan, CP - Crystallizer pan, B – Brine and S - Sediment

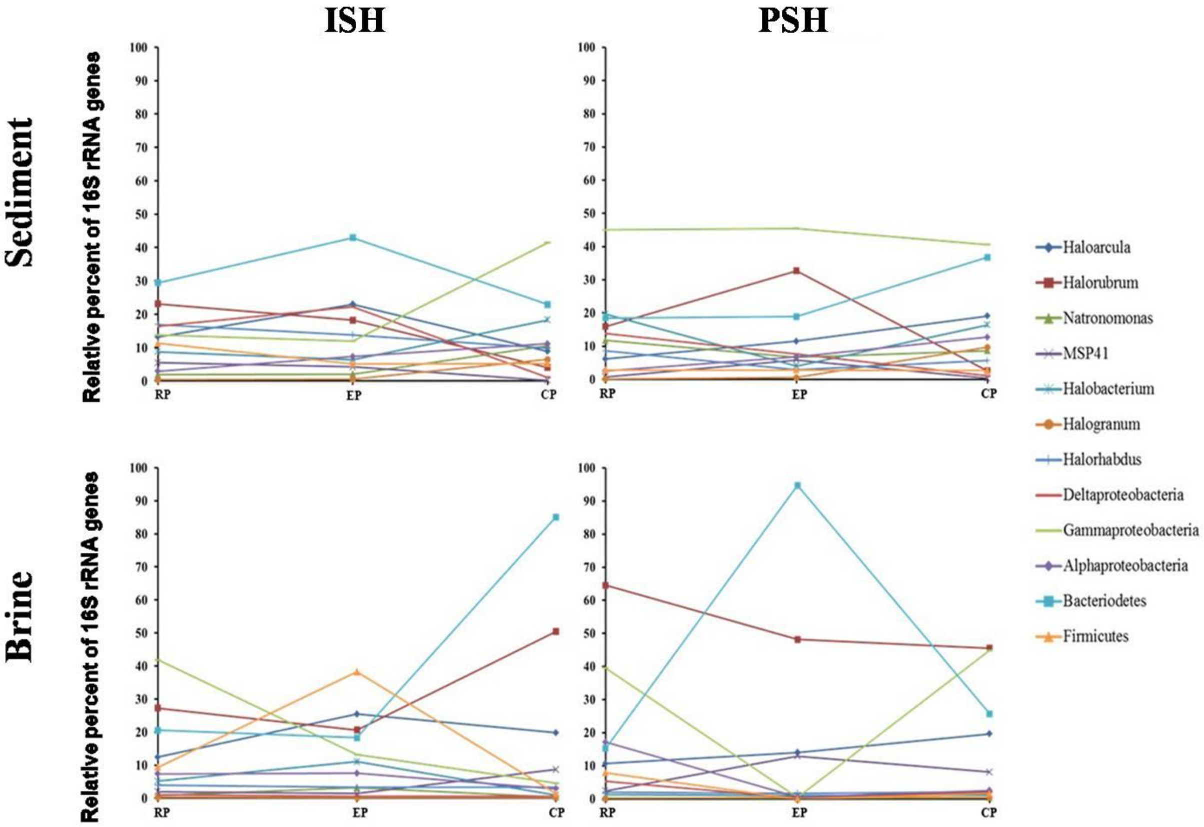

Supplementary Figure 4: Line charts describing the distribution of dominant microbial members through increasing salinity gradient from RP to CP during ISH and PSH in sediment and brine.
